# Olive Oil Injection in Typhoid Fever

**Published:** 1900-12-15

**Authors:** 


### OLIVE OIL INJECTION IN TYPHOID FEVER.

In these days of medical scepticism it is extremely pleasing to find a physician who is thoroughly satisfied with his power over disease. Dr. Owen Paget, of Fremantle, Western Australia, seems to be a good representative of the hopeful class of practitioners. Writing in the *Lancet* on the subject of the treatment of typhoid fever, he strongly advocates the employment of injections of olive oil per rectum, the quantity to be used being about a pint at a time, the injections being administered every twelve to twenty-four hours, and the oil being retained in the bowel from twelve to twenty-four hours if possible. If not returned in this time it may be brought away by an ordinary soap and water injection, and a fresh dose of oil administered two or three hours later. After a week or ten days the daily use of the injections can be discontinued, and they may be given only when the temperature rises or the bowels are confined. So impressed with the efficacy of this method of treatment is Dr. Paget that he holds that "patients treated in this manner, in my opinion, cannot die; for careful observation has led me to believe that typhoid *per se* is a harmless fever; it is the accompanying sapræmia or ptomaine poisoning which produces the ill-effects. Under this line of treatment there are no sequelæ, tympanites, perforations, or heart failure. No cold baths are necessary. The most unskilled nursing is sufficient." Here is faith! No one, after this, can say that the profession is eaten up by an all-pervading scepticism and distrust in its own remedies. Still, putting on one side the extravagances of an over-confident man, we have no doubt that Dr. Paget has a certain basis for what he says, for there is every reason to believe that much more can be done for typhoid fever than is included in the mere "rest and feed" programme which is so commonly followed at the present day. He is certainly right in regard to the large part played by sapræmia in the production of the "typhoid state," and there can be but little doubt that it is often a far better plan to remove offensive matters as he does by their appointed route than to make the elaborate attempts at intestinal antisepsis to which some have so largely devoted their energies. To say, however, that patients treated in this manner "cannot die," is to bring ridicule on a proceeding which might be useful in many cases.

